# MEMORY CHANGES IN COGNITIVELY HEALTHY OLDER ADULTS: AGE EFFECTS IN MEMORY-BASED INFERENCE

**DOI:** 10.1093/geroni/igae098.2257

**Published:** 2024-12-31

**Authors:** Cara Charles, Ra’Shida Rockette, Caitlin Bowman

**Affiliations:** University of Wisconsin Milwaukee, Milwaukee, Wisconsin, United States; UWM, Milwaukee, Wisconsin, United States; University of Wisconsin Milwaukee, Milwaukee, Wisconsin, United States

## Abstract

Memory changes in older age are well documented, even among cognitively healthy older adults. Most prior research has focused on age-related declines in remembering details of individual experiences. However, memory-based inference is another important memory ability that allows us to make connections across separate experiences to make novel decisions. Little is known about the extent of age differences in inference abilities. In the present experiment, we administered an inference task to young and cognitively healthy older adults that asked them to both remember pairs of items that were presented directly (e.g., apple – truck, truck - clock) and that could be combined to form an indirect inference association that was not shown (e.g., apple-clock). Test results showed above-chance performance in older adults for both remembering direct pairs and making inferences. However, older adults performed worse than young adults overall, with a larger age deficit in inference compared to direct memory. We also tested for age differences in the ability to remember which context the associations were learned in (direct vs. indirect) because a loss of context memory can indicate separate experiences have been combined in memory. Successful inference was more likely to lead to a loss of context memory in older adults compared to young adults, but further analyses indicate that strategic responding in older adults could account for this difference. Overall, we found that older adults had an additional cost to forming inferences compared to remembering direct items, but the underlying mechanisms of that difference will require future investigation.

